# Conserved queen pheromones in bumblebees: a reply to Amsalem et al.

**DOI:** 10.7717/peerj.3332

**Published:** 2017-05-16

**Authors:** Luke Holman, Jelle S. van Zweden, Ricardo C. Oliveira, Annette van Oystaeyen, Tom Wenseleers

**Affiliations:** 1 School of Biosciences, University of Melbourne, Melbourne, Victoria, Australia; 2 Laboratory of Socioecology and Social Evolution, Zoological Institute, University of Leuven, Leuven, Belgium; 3 Research and Development, Biobest Belgium NV, Westerlo, Belgium

**Keywords:** Eusociality, Cuticular hydrocarbons, Fertility signals, Reproductive division of labour

## Abstract

In a recent study, Amsalem, Orlova & Grozinger (2015) performed experiments with *Bombus impatiens* bumblebees to test the hypothesis that saturated cuticular hydrocarbons are evolutionarily conserved signals used to regulate reproductive division of labor in many Hymenopteran social insects. They concluded that the cuticular hydrocarbon pentacosane (C_25_), previously identified as a queen pheromone in a congeneric bumblebee, does not affect worker reproduction in *B. impatiens*. Here we discuss some shortcomings of Amsalem et al.’s study that make its conclusions unreliable. In particular, several confounding effects may have affected the results of both experimental manipulations in the study. Additionally, the study’s low sample sizes (mean n per treatment = 13.6, range: 4–23) give it low power, not 96–99% power as claimed, such that its conclusions may be false negatives. Inappropriate statistical tests were also used, and our reanalysis found that C_25_ substantially reduced and delayed worker egg laying in *B. impatiens*. We review the evidence that cuticular hydrocarbons act as queen pheromones, and offer some recommendations for future queen pheromone experiments.

## Introduction

Over the last 20 years, evidence has accumulated that specific cuticular hydrocarbons (CHCs), which consistently differ between fertile and non-fertile colony members, help to regulate reproductive division of labour in eusocial ants, bees and wasps (e.g., Monnin, Malosse & Peeters, 1998; Liebig et al., 2000; Dietemann et al., 2003). This theory originally rested on indirect evidence, including observations that queens and workers apparently always differ in their CHC profiles (reviewed in Van Oystaeyen et al., 2014), that CHC profiles correlate with inter-individual variation in fecundity within a given caste (e.g., D’Ettorre et al., 2004; Holman, Dreier & D’Ettorre, 2010), and that workers can discriminate between the CHCs of fertile and non-fertile individuals (Dietemann et al., 2003; D’Ettorre et al., 2004). Recently, studies using synthetic hydrocarbons have experimentally demonstrated that queen-like CHCs affect worker ovarian development (in seven species; Van Oystaeyen et al., 2014; Holman et al., 2010; Holman, Lanfear & D’Ettorre, 2013; Holman, Hanley & Millar, 2016; Holman, 2014; De Narbonne et al., 2016; Oi et al., 2016), and/or induce behavioural changes in workers that are putatively related to reproduction (in three species; Holman et al., 2010; De Narbonne et al., 2016; Smith, Millar & Suarez, 2015; Smith, Hölldobler & Liebig, 2009). A recent comparative analysis of chemicals thought to be correlated with caste or fertility in 64 species of social Hymenoptera concluded that these chemicals were most commonly saturated CHCs, and that the correlation between saturated CHCs and female fecundity appears to be ancestral in Hymenoptera (Van Oystaeyen et al., 2014). Because eusociality evolved several times in this clade, this result suggests that queen pheromones evolved from chemical signals or cues that were already present in the solitary common ancestor of bees, ants and wasps, which lived *c.* 145 million years ago (Van Oystaeyen et al., 2014).

Recently, Amsalem, Orlova & Grozinger (2015) performed bioassays with synthetic hydrocarbons in *Bombus impatiens* bumblebees to test the hypothesis that saturated CHCs are evolutionarily conserved signals used to regulate reproductive division of labour. In the congeneric bumblebee *B. terrestris*, two earlier experiments concluded that workers resorbed oocytes more often (Van Oystaeyen et al., 2014) and had fewer developing oocytes in their ovaries (Holman, 2014) after treatment with the queen-characteristic cuticular hydrocarbon pentacosane (C_25_), leading those studies to conclude that C_25_ was a queen pheromone. Amsalem et al. reported that worker reproduction was unaffected by C_25_, and also not affected by two other cuticular hydrocarbons referred to as “controls” (C_23_ and C_27_—though these CHCs also correlated with fecundity, and so should perhaps instead be regarded as putative queen pheromones). Because the three hydrocarbons had no statistically significant effect on worker reproduction, Amsalem et al. concluded that saturated hydrocarbons associated with fertility do not affect worker reproduction in *B. impatiens*, and suggested that the theory presented above be reconsidered.

Although we are excited to see new experimental data in this area, we feel that Amsalem et al.’s conclusions are not justified by their data. We first point out some methodological problems with the study, then present a statistical reanalysis of its data. We conclude that the study does not conclusively demonstrate that the three fertility-linked hydrocarbons C_23_, C_25_ and C_27_ are not pheromones, as it claimed. Although confounding effects make the data difficult to interpret, the new results tentatively suggest that worker fecundity is reduced following exposure to queen CHCs. We conclude with some suggestions for the design of future experiments.

## Methodological Issues

**Figure 1:**
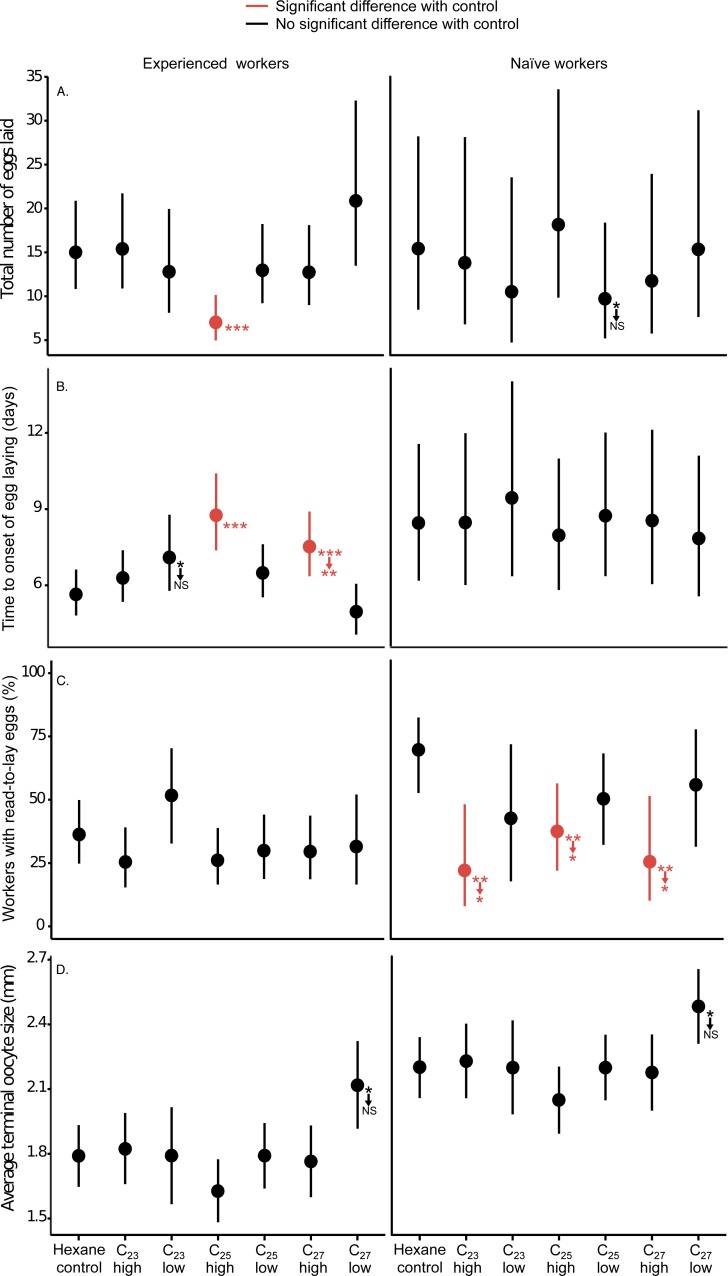
The least-square means (and their 95% confidence limits) for each hydrocarbon treatment, calculated from models shown in Tables S4–S7. Significant 2-tailed differences with the appropriate hexane control in planned contrasts are indicated using asterisks (^∗∗∗^, *p* < 0.001, ^∗∗^, *p* < 0.01, ^∗^, *p* < 0.05; NS, *p* > 0.05). Results for which the significance level changed following Benjamini–Hochberg false discovery rate correction are indicated with downward arrows pointing to the new significance level. The results of corresponding fixed effect GLMs and Bayesian GLMMs were largely concordant and are presented in Tables S4–S7.

We believe there are three methodological shortcomings in the new study. Firstly, Amsalem et al. aimed to test whether workers’ responses to queen pheromones involve learning. They did this by examining responses to synthetic hydrocarbons in both “experienced” and “naïve” workers, so-called because the experienced workers had spent more time in a nest containing a live queen. However, the experienced/naïve treatment was confounded, and we believe that it provides only limited information about the role of learning in the response to queen CHCs. Amsalem et al. did not take the conventional experimental approach of starting with a common pool of individuals and then randomly dividing them between the two learning treatments, but rather used two different sets of workers to set up the two treatments. As a result, the naïve workers were younger and larger, which likely explains why they had larger oocytes and a longer latency to egg laying irrespective of CHC treatment. This means that any effect of the naïve/experienced treatment on the responsiveness to queen CHCs could be due to differences in age, size or reproductive physiology rather than learning. Moreover, the colony fragments of experienced workers contained three workers from the same colony, while the naïve colony fragments contained workers from an unspecified mixture of one, two or three different colonies. The effect of exposure to foreign vs same-colony workers on the response to queen pheromone is untested, so this might be a problem. Finally, the sample size in the naïve treatment was twofold lower than in the experienced treatment (Table 1). This means that the differences in p-values between experienced and naïve bees may reflect a difference in statistical power (as reflected in the differing widths of the confidence intervals in our Fig. 1), rather than lower responsiveness in the naïve bees, as was claimed.

**Table 1:** Sample sizes in Amsalem et al.’s experiment. The table highlights that sample sizes were low and uneven, that certain colonies are over-represented in particular hydrocarbon treatments, and that the naïve and experienced treatments used mixed-colony or single-colony groups of workers, respectively. Note that we give the sample size in terms of the number of colony fragments, which is appropriate for the colony-level variables ‘egg number’ and ‘latency to egg laying’. For response variables measured at the level of individual workers (i.e., presence of ‘ready-to-lay’ eggs, length of terminal oocycte, and oocyte resorption) the sample sizes are c. 3-fold higher, because each colony fragment contained three workers.

		Colony								Total *n*
		a	b	c	d	e	f	g	Mix of 1–3 colonies	
Control	Experienced	8	2	–	3	1	3	6	–	23
C_23_ High	Experienced	8	–	–	2	4	2	4	–	20
C_23_ Low	Experienced	–	–	–	–	5	1	4	–	10
C_25_ High	Experienced	10	–	–	4	4	2	3	–	23
C_25_ Low	Experienced	8	1	1	1	4	1	5	–	21
C_27_ High	Experienced	7	–	–	3	4	2	4	–	20
C_27_ Low	Experienced	–	–	–	–	4	2	4	–	10
Average		8.2	1.5	1	2.6	3.7	1.9	4.3		18.1
Control	Naïve	–	–	–	–	–	–	–	16	16
C_23_ High	Naïve	–	–	–	–	–	–	–	6	6
C_23_ Low	Naïve	–	–	–	–	–	–	–	4	4
C_25_ High	Naïve	–	–	–	–	–	–	–	13	13
C_25_ Low	Naïve	–	–	–	–	–	–	–	12	12
C_27_ High	Naïve	–	–	–	–	–	–	–	6	6
C_27_ Low	Naïve	–	–	–	–	–	–	–	6	6
Average									9.0	9.0

Secondly, the allocation of workers to CHC treatments was imbalanced such that different hydrocarbon treatments contained workers from different colonies (Table 1). This unequal allocation could have biased the results, because colonies differed in body size (Table S1A), which in turn produced significant differences in body size between the seven hydrocarbon treatments (Table S1B). Because body size correlated with most of the variables under study (Tables S4–S8), the allocation of different-sized workers from various colonies to particular treatments may have skewed the results.

Thirdly, the ‘Low’ pheromone dose used in Amsalem, Orlova & Grozinger (2015) is very low relative to all previous queen pheromone experiments (see Table S2 for a review), and might be artificially low relative to natural conditions. Amsalem et al. chose not measure the mass of CHCs produced by queen or worker *B. impatiens*, and so it is unclear whether or not their experiment used a natural dose. Therefore, we analysed the cuticular hydrocarbon profiles of *B. impatiens* queens and workers using GC-MS (see Supplementary Methods), to obtain this information. We found that the ‘Low’ doses in Amsalem et al. represented approximately one ten-thousandth of the mass of CHCs found on the cuticle of a live queen (Table S2). In terms of “queen equivalents”, this is approximately 140 × lower than in Holman (2014) and 33, 000 × lower than the doses used in Van Oystaeyen et al. (2014) (Table S2), which may explain any difference in results between Amsalem et al. and the studies it set out to challenge. Although we acknowledge that it is difficult (if not impossible) to identify a biologically meaningful dose of queen pheromone in this type of experiment, we propose that the unusually low CHC concentration (especially in the “Low” treatment) may explain some of the new paper’s null results.

## Statistical Issues

Firstly, Amsalem, Orlova & Grozinger (2015) applied classical ANOVA to types of data that can violate ANOVA’s assumptions, including censored time series data and count data, which do not match the expected theoretical distributions and do not allow for appropriate bounds on the measurement scale. To address this, we instead analysed the data using generalized linear or generalized linear mixed models (GLM and GLMM) that make distributional assumptions matching those that are theoretically expected (e.g., Poisson models for count data) and which respect the bounds of the measurement scale by using an appropriate link function (e.g., the log link used in Poisson models ensures that counts are strictly positive). For time series data, we used survival models that appropriately model censoring in the data.

Secondly, Amsalem et al. searched for a significant difference between all possible pairwise combinations of the seven CHC treatment groups for multiple different metrics of worker reproduction, which results in a very large number of post-hoc tests. Because the authors corrected for multiple testing, the excessive number of tests greatly reduced statistical power. To address this problem, we used planned contrasts to compare each of the 6 CHC treatments to their respective control (i.e., 12 tests per ovary metric, because of the experienced/naïve treatment). Comparisons between a CHC and the control group are most informative for testing whether that CHC induces worker sterility—it is less interesting to test, for example, whether the low dose of C_23_ had a different effect to the high dose of C_27_.

Thirdly, most of Amsalem et al.’s analyses do not statistically account for the non-independence introduced by the use of workers derived from the same colonies (or colony fragments, for the individual-level response variables), meaning that their analyses incur pseudoreplication. One exception appears in Table S7 of Amsalem, Orlova & Grozinger (2015), in which the authors fit ANOVAs with CHC treatment, colony, and their interaction as fixed effects. Because of the imbalanced experimental design, the authors chose to discard all the data from colonies a-d (i.e., 46% of the dataset; Table 1) in order to fit the treatment × colony interaction. If one instead uses all the data (which necessitates omitting the interaction term), one recovers the significant CHC treatment effects on worker egg laying, latency to egg-laying and worker ovary activation that we report below.

Fourthly, as mentioned above, worker body size differed substantially across pheromone treatments (Tables S1A and S1B), likely because of non-random treatment allocation, which is problematic because fecundity correlates with body size (Tables S4–S8). We therefore decided to statistically correct for this confounding difference in body size by including it as a covariate in our analyses (however, omitting body size did not qualitatively change the results we present here). Our models thus estimate the effect of treatment on the body size-corrected response variables.

Amsalem et al. reported on four response variables pertinent to the hypothesis that queen hydrocarbons affect worker reproduction: number of eggs laid (over 10 days), days until the onset of egg laying (censored after day 10), frequency of oocyte resorption, and mean size of the terminal oocytes. To facilitate comparison with past queen pheromone experiments, many of which treat ovary activation as a binary variable, we also coded a fifth response variable that was discussed in Amsalem, Orlova & Grozinger (2015) but not formally analysed: the frequency of workers with fully activated ovaries. We define this variable as the frequency of workers with ovaries in which the largest oocytes were >2 mm long (described as “‘ready to lay’ eggs” in Amsalem, Orlova & Grozinger (2015)), and in which the terminal oocyte was not resorbed (because resorption suggests that the worker will not lay a viable egg soon; Duchateau & Velthuis, 1989). This metric is also directly comparable to the one used in Van Oystaeyen et al. (2014), which is one of the past studies that Amsalem et al. were following up. As evidence that this metric is biologically meaningful, we found that it was a good predictor of egg laying, and was a better predictor than other possible binary measures of ovary activation derived from Amsalem et al.’s dissection data (Table S3).

## Results of Our Statistical Analysis

Our reanalysis found evidence that one or more hydrocarbons significantly inhibited worker reproduction, for some but not all of the ovary metrics (Fig. 1; Tables S4–S8). Additionally, our reanalysis does not support claims in Amsalem, Orlova & Grozinger (2015) that the experiment had “a power of 99% and 96% for effect size of 0.2 and 0.5, respectively” (sic) to detect treatment effects. For example, Fig. 1 shows that the 95% confidence intervals are large, such that many of the non-significant results are consistent with large treatment effects. We have archived Amsalem et al.’s raw data, and the R scripts for our new analyses, alongside this article.

Following requests from reviewers, we analysed each dataset with multiple different modelling approaches, in order to verify that our results are robust to the choice of model used. For example, we investigated the egg count data in Fig. 1A with Poisson generalised linear models (GLM; colony was modelled as a fixed effect), Poisson generalised linear mixed models (GLMM; colony modelled as a random effect), and a Bayesian Poisson generalised mixed model (see Tables S4–S8 and attached R script for full details). In every case, the qualitative conclusions were highly concordant, suggesting the differences with the conclusions in Amsalem, Orlova & Grozinger (2015) are not particular to some cherry-picked model. For brevity, Fig. 1 shows the results of our preferred analyses only (i.e., frequentist GLMMs and a mixed effects survival analysis) and omits the one response variable (oocyte resorption) for which there was no qualitative difference between our results and those in Amsalem, Orlova & Grozinger (2015) (namely, that all three queen CHCs induced significantly higher oocyte resorption).

### Amount and timing of worker egg laying

Egg number and the latency to egg laying are arguably the most direct measures of worker reproduction. Amsalem et al. reported no effect of hydrocarbons on either variable using ANOVA and post-hoc testing. Our reanalysis suggested that the high dose of C_25_ caused worker groups to lay half as many eggs, and to take 55% longer to begin egg laying, relative to the control, in experienced workers (Poisson GLMM and mixed effects survival analysis—results agreed with GLM and Bayesian GLMM models; Figs. 1A–1B; Tables S4–S5). In addition, the C_27_-high treatment delayed the onset of egg laying in experienced workers (Fig. 1B). All of these results remained significant after controlling for multiple testing (Benjamini & Hochberg, 1995).

### Frequency of workers with fully activated ovaries

Among naïve workers, all three of the high-dose hydrocarbon treatments significantly reduced the proportion of workers with activated ovaries (binomial GLMM plus alternative analyses; Fig. 1C; Table S6). The effect size of hydrocarbons on the frequency of workers with active ovaries was of similar magnitude to that observed in previous comparable experiments in bumblebees, wasps and ants (but not honeybees) using analogous response variables (Table S2). All of these results remained significant after controlling the false discovery rate (Benjamini & Hochberg, 1995).

We speculate that the ‘experienced’ workers responded to the queen hydrocarbons with a reduction in egg laying, while the ‘naïve’ workers responded with a reduction in ovary activation only towards the end of the experiment, because of confounding differences in the age of these workers. The naïve workers all had undeveloped ovaries at the start of the experiment (since these workers were <24 h old), while the experienced workers were presumably in a variety of stages of reproductive development, priming them to begin egg laying sooner (as was observed: Fig. 1 and Amsalem, Orlova & Grozinger, 2015), and making the effects of pheromones on egg number more pronounced in the ‘experienced’ treatment (since egg laying occurred over more days in the experienced treatment than in the naïve treatment).

### Mean size of terminal oocytes in workers’ ovaries

Hydrocarbon treatment did not significantly affect the size of the terminal oocytes. Workers receiving the ‘High’ dose of the *B. terrestris* queen pheromone C_25_ had non-significantly smaller terminal oocytes than the control (*p* = 0.077 in a planned contrast, i.e., uncorrected for multiple testing; Figs. 1D; Table S7).

### Frequency of oocyte resorption

We also replicated Amsalem et al.’s finding that all three hydrocarbons induced significantly more oocyte resorption than the hexane control (binomial GLMM; Table S8; not shown in Fig. 1 since the results are the same as in Amsalem, Orlova & Grozinger (2015)). Although this finding is consistent with the three CHCs somehow affecting ovaries, the biological significance is harder to interpret, because oocyte resorption in *B. impatiens* had a weak *positive* relationship with egg-laying (Amsalem, Orlova & Grozinger, 2015). This result contrasts with a previous study of a different *Bombus* species, in which oocyte resorption was more common in workers living in queenright rather than queenless colonies (Table S2D in Van Oystaeyen et al. (2014)), suggesting that queenless workers begin to reproduce because they cease resorbing their oocytes.

### Effect of body size on measures of worker reproduction

In addition to the large effect of colony identity seen in most of the analyses (Table S3), worker body size was confirmed to have a significant effect on worker egg-laying and ovary development in nearly all analyses (Tables S4–S8). Specifically, cages with larger workers produced more eggs (*p* = 0.001) and laid earlier (*p* = 0.0003), and larger workers had bigger oocytes (*p* < 0.0001) and were more likely to display oocyte resorption (*p* = 0.03).

These results underscore the importance of controlling for body size, either experimentally or statistically, when studying reproduction in size polymorphic insects such as bumblebees. Out of curiosity, we re-analysed the data from our previous bumblebee queen pheromone experiment (Van Oystaeyen et al., 2014) after including body size as a covariate, in order to check the conclusions of Van Oystaeyen et al. (2014) were robust. The treatment effect of C_25_ on oocyte resorption remained, and we found that larger workers were less likely to have resorbed oocytes (Table S9).

## Conclusions and Recommendations

To conclude, we suggest that Amsalem et al.’s claim—that their experiment definitively demonstrates that three fertility-associated hydrocarbons do not reduce worker reproduction in *B. impatiens*—does not follow from their data. The experiment has an unbalanced and comparatively low sample size, which is problematic because the study’s conclusion rests upon its failure to reject the null hypothesis. We also highlighted a number of methodological problems, such as confounding effects caused by non-random assignment of individuals to treatments, which complicate interpretation of the data.

Our reanalysis found evidence that C_25_, the same queen pheromone identified in *B. terrestris* (Van Oystaeyen et al., 2014; Holman, 2014), substantially reduced the number of eggs laid, delayed the onset of laying, and reduced the frequency of workers with activated ovaries in *B. impatiens*. We also found limited evidence that the other two fertility-associated hydrocarbons (C_23_ and C_27_) might perform a similar function. The results are patchy (Fig. 1), and our reanalysis is not decisive because of the issues with the data. Nevertheless, the reanalysis makes it clear that the new study does not comprehensively reject the hypothesis that queen-like CHCs are not involved in regulating reproduction in *B. impatiens* workers, as claimed.

We suggest the following modifications to future experiments to help ensure reliable results. Firstly, an appropriate sample size is needed to ensure adequate statistical power, particularly when the effect sizes are expected to be moderate (see effect size estimates from past queen pheromone experiments in Table S2). Although we applaud the effort in Amsalem, Orlova & Grozinger (2015) to examine multiple chemicals, doses, and categories of workers, the workload needed to maintain an adequate sample size becomes prohibitive very quickly, and so it may be better to design experiments with good replication but fewer treatments. Secondly, one should start with a common pool of individuals and then randomly allocate them to treatments, rather than allocating different pools (e.g., young and old workers, or big and small workers) to different treatments, producing confounding effects. This can be done by splitting colonies randomly and equally between pheromone treatments (as in e.g., Van Oystaeyen et al., 2014; Holman et al., 2010; Holman, Lanfear & D’Ettorre, 2013; Holman, Hanley & Millar, 2016; De Narbonne et al., 2016; Oi et al., 2016), or randomly assigning whole colonies to different treatments (e.g., Van Oystaeyen et al., 2014; Holman, 2014). It is also important to run the different experimental treatments in parallel, rather than running one treatment and then another, such that environmental factors or cohort effects could confound the results (it is unclear whether this was done in Amsalem, Orlova & Grozinger (2015), but the differences in sample size and worker colony origin imply that it was not). Thirdly, we acknowledge that it can be difficult to select the correct dose of pheromone in this type of study, since we can think of no foolproof way to accurately measure the dose to which workers are exposed in natural colonies. The ideal experiment may be to compare worker responses to natural queens, queens whose pheromone was somehow selectively removed) e.g., through using genetic manipulation), and appropriate control queens. Korb et al. (2009) performed such an experiment, in which they used RNAi to knock out a gene putatively involved in chemical communication in queen termites, and observed an increase in a worker behavior associated with queenlessness. The challenge for such experiments is to ensure that the only change in the queen is the removal of her pheromone. Alternatively, one could test multiple doses of pheromone that span the conceivable range of concentrations that workers might experience. Finally, one could test whether queen pheromones are learned by collecting naïve workers, giving them a ‘training phase’ with either no queen, a queen of low fecundity, or a queen of high fecundity, and then later measuring their physiological or behavioral responses to queen pheromone.

##  Supplemental Information

10.7717/peerj.3332/supp-1 Supplemental Information 1 Supplementary methods, and table legends for supplementary tables

10.7717/peerj.3332/supp-2 Supplemental Information 2 Supplementary tables See associated PDF for the table legends.

10.7717/peerj.3332/supp-3 Supplemental Information 3 R script to replicate our statistical reanalysis See annotations inside the script.

10.7717/peerj.3332/supp-4 Supplemental Information 4 Raw data from Amsalem et al. on group-level measures of reproduction Raw data in a .csv file.

10.7717/peerj.3332/supp-5 Supplemental Information 5 Raw data from Amsalem et al. on individual-level measures of reproduction Raw data in a .csv file.

10.7717/peerj.3332/supp-6 Supplemental Information 6 Raw data from Van Oystaeyen et al. with body size also included Needed to perform our reanalysis of this data, including body size.

## Additional Information and Declarations

### Data Availability

The following information was supplied regarding data availability: The raw data has been supplied as a Supplemental Dataset.
