# Supplementary material for: Conserved queen pheromones in bumblebees: a reply to Amsalem et al
Source: PeerJ. 2017 May 16;5:e3332. doi: 10.7717/peerj.3332 (PMC5436554; doi:10.7717/peerj.3332)
Supplement: Supplemental Information 1 [file peerj-05-3332-s001.docx]

**Supplementary Methods**

To estimate the amount of cuticular compounds of *Bombus impatiens*, we collected queens and workers from ten colonies obtained from BioBest Canada Ltd. (Leamington, Ontario, Canada). CHCs were extracted by immersing individual bumble bees in HPLC-grade pentane (Sigma-Aldrich, Belgium), 5 ml in case of queens or 2 ml in case of workers, for 5 min with vortexing at 600 rpm for 10 sec at the start and the end. The pentane was then evaporated, and all extracts were reconstituted in 1 ml HPLC-grade hexane (Sigma-Aldrich, Belgium), and transferred to 2 ml vials. We then injected 0.5 µl of extract into a Thermo Scientific TRACE 1300 gas-chromatograph coupled with a Thermo Scientific ISQ mass-spectrometer. Injection was done in split mode (10:1), using an injection temperature at 320 °C, MXT-5 stainless steel capillary column (30 m × 0.25 mm × 0.25 µm), helium as the carrier gas at 0.9 ml/min, a MS transfer-line temperature of 300 °C, and an ion source temperature of 300 °C. The oven temperature was held at 40 °C for 2 min, then increased to 120 °C at 20 °C/min, then to 200 °C at 10 °C/min, then to 250 °C at 7 °C/min, and then to 350 °C at 5 °C/min, with a final hold of 15 min at 350 °C. AMDIS 2.71 in combination with NIST MS Search 2.2, NIST 2014 library search, and manual interpretation of mass spectra (taking into account expected fragmentation patterns and retention indices) were used to identify the different compounds. Peak areas of 29 hydrocarbons were integrated using a custom script (available from the authors upon request) in R v3.2.4. Retention indices and absolute quantities of our compounds were calculated using cubic spline interpolation based on the elution times and peak areas of an external n-C7 to n-C40 linear alkane ladder standard (49452-U, Supelco) that was run on the same program with concentrations of 0.1, 0.01 and 0.001 µg/µl.

The results of this analysis can be found in Supplementary Table S11.

**Legends for supplementary tables**

*Table S1*: ANOVA analyses demonstrating that worker body size significantly differed across colonies and across the hydrocarbon and naivety treatments, and therefore should be included as a covariate in subsequent analyses to minimise confounding effects.

*Table S2*: Overview of the methods and results of experimental studies investigating the effects of synthetic putative queen pheromones on worker ovaries or reproduction. In all cases, the original data was requested (allowing us to recalculate effect size in a standardized way – from a GLMM with colony as a random factor), but if these were not available the frequencies of workers with developed ovaries were estimated from the published papers. The papers were found using an exhaustive search on Google Scholar.

*Table S3*: Analysis to find the ovary metrics that best predict the amount of worker egg-laying. Table S3a gives the results of a quasi-Poisson GLM with colony treated as a fixed factor, whereas Table S3b gives those of a Poisson generalized linear mixed model, where cage ID and colony ID are treated as random factors in the model (the cage ID random effect was included to correct for overdispersion). In both cases, the proportion of workers with developed and non-resorbed, developed and resorbed, and non-developed and resorbed ovaries were included as covariates, whilst correcting for the total number of days over which eggs were laid in each cage. The fourth category (i.e. non-developed and non-resorbed) was left out to prevent the model from becoming rank deficient, since the proportion of workers in each category sums to one. Workers with developed ovaries were scored as those with a mean terminal oocyte size of >2 mm. The proportion of workers with developed and non-resorbed oocytes was the strongest predictor of actual egg-laying.

*Table S4*: Results of three different models of the total number of eggs produced over 10 days in cages containing 3 workers. Tables S4a-c give the results from a Poisson GLMM with colony treated as a random factor (allowing all the data to be analysed in a single model), the experienced/naïve treatment as a fixed factor and mean-centered group mean body size as a covariate. Tables S4d and S4e describe the results of a pair of quasi-Poisson GLMs, which separately analyse the data for the naïve and experienced workers (splitting the dataset like this was necessary because colony was confounded with the experience treatment). Colony and treatment were entered as fixed effects, and mean-centered group mean body size was included as a covariate. Finally, Table S4f gives the results of a Poisson GLMM conducted in a Bayesian framework using the *brms* package for R. The results of the three modeling approaches are all broadly concordant: all three models suggest that C_25_ significantly reduced the number of eggs laid relative to the hexane control. All models also showed that cages that contained larger workers laid significantly more eggs.

*Table S5*: Tables S5a-c describe the results of an analysis of the latency for workers to begin laying eggs based on a Weibull frailty model with treatment, worker type and treatment × worker type as fixed effects, mean-centered group mean body size of the workers as a continuous covariate, and colony ID as a Gaussian frailty term. The tables show the summary coefficient table, least square means for treatment and worker type, and the response ratio for each hydrocarbon treatment relative to the hexane control (based on posthoc tests with FDR correction of *p* values for multiple testing). Tables S5d-S5e show the results of a similar model but with colony entered as a fixed factor instead of a frailty term. In this case, however, separate models were run for naïve and experienced workers because colony and experienced were confounded and the model could not handle this elsewise. Table S5f shows the results of a pair of Bayesian proportional hazards models (one for naïve workers, one for experienced workers), fitting the effects of hydrocarbon treatment and mean body size, as well as a Gaussian frailty term (fit using the using the *brms* package for R). Both the frequentist and Bayesian models found that C_25_ significantly increased the time taken by workers to begin laying eggs, and that cages with larger workers started laying eggs earlier.

*Table S6*: Results of three different models of the frequency of workers with active ovaries, which as in [4] we defined as containing ready-to-lay eggs in their ovaries (i.e. containing fully mature oocytes with a terminal oocyte size >2 mm with no signs of oocyte resorption). Tables S6a-c show the results of a binomial GLMM with treatment, worker type and treatment × worker type as fixed effects, mean-centered worker body size as a continuous covariate, and cage ID and colony ID as random intercepts. Results show the summary coefficient table, least square means for treatment and worker type, and the response ratio for each hydrocarbon treatment relative to the hexane control (based on posthoc tests with FDR correction of *p* values for multiple testing). Tables S6d-e show the results of a pair of binomial generalized linear models (one for experienced workers, one for naïve workers; as before, the data needed to be split because colony and treatment were confounded) in which treatment and colony were included as fixed factors and mean-centered body size as a covariate. Table S6f shows a binomial GLMM conducted in a Bayesian framework using the *brms* package for R. Finally, Tables S6g-h and S6i show the results of a fixed-effect and of a random-effect ordinal logistic regression in which the 4 possible ovary development scores were analyzed on an ordinal scale (in increasing order of ovary development: 1=non-developed and non-resorbed, 2=non-developed and resorbed, 3=developed and resorbed and 4=developed and non-resorbed). The results of all five modeling approaches are all broadly concordant, and both the Bayesian and frequentist binomial mixed models as well as the ordinal models suggest that the three “high” treatments reduced the frequency of naïve workers with active ovaries.

*Table S7:* Analysis of the mean size of the terminal oocytes in the workers’ three largest ovarioles. The distribution of these data is truncated (since an egg cannot be smaller than zero, nor much larger than an average-sized mature egg) and exhibits bimodality, so we rescaled the oocyte size data to lie between 0 and 1 and then analyzed them using a beta-distributed generalized additive mixed model with logit link function. We selected the most parsimonious model using the Aikaike Information Criterion, which contained treatment, worker type (experienced or naïve) and mean-centered worker body size (but not the treatment-type interaction); we also included cage ID and colony ID as random effects in all models compared. Results show the Type III ANOVA table, the summary coefficient table (which shows contrasts with the hexane control), and the least square means for treatment and worker type.

*Table S8:* Analysis of the proportion of workers displaying oocyte resorption. The model is a binomial GLMM with treatment, worker type and treatment × worker type as fixed effects, mean-centered worker body size as a continuous covariate, and cage ID and colony ID as random intercepts. Results show the summary coefficient table, least square means for treatment and worker type, and the response ratio for each hydrocarbon treatment relative to the hexane control (based on posthoc tests with FDR correction of *p* values for multiple testing). Analysing the data with Bayesian GLMM or GLM yielded qualitatively identical results. These results are not illustrated in Fig. 1, since our conclusions concur with Amsalem et al.’s for these data.

*Table S9:* Reanalysis of the queen pheromone bioassays of Van Oystaeyen et al. [4] on the proportion of workers displaying oocyte resorption or having active ovaries in *B. terrestris* using binomial GLMMs with explicit consideration of worker body size (which was not considered in [4] but which we have measured since). As above, active ovaries were defined as containing ready-to-lay eggs (i.e. with a mature terminal oocyte with no signs of oocyte resorption; ovary development scale IV of [18]). The models used are binomial GLMMs with treatment included as a fixed factor and mean-centered colony size and mean-centered worker body size (thorax width) as covariates, and with colony ID as a random intercept. Results show the summary coefficient table and the response ratio for each hydrocarbon treatment relative to the hexane control (based on posthoc tests with FDR correction of *p* values for multiple testing). Results confirm the previously reported effect that C_25_ causes a significant increase in oocyte resorption [4], which in this species has been shown to be linked to suppression of worker reproduction [4], and that larger workers are significantly more likely to have active ovaries and less likely to display oocyte resorption.

*Table S10:* Reanalysis of the queen pheromone bioassays of Holman [10] of the the number of visible oocytes present in the workers’ ovaries and of the proportion of workers having developed ovaries (defined as containing more than 10 visible oocytes) in *B. terrestris* using negative binomial and binomial GLMMs and with explicit consideration of worker body size (here simply coded as small, medium or large). The models used are binomial or negative binomial GLMMs with treatment included as a fixed factor and mean-centered worker body size as a covariate, and with colony ID as a random intercept. Results show the summary coefficient table of each analysis (here no FDR correction of *p* values for multiple testing was required as there was only one treatment). Results confirm the previously reported effect that C_25_ causes a significant decrease in visible oocyte number [10] and a decrease in the probability for workers to have developed ovaries, and also show that larger workers had more visible developing oocytes in their ovaries and were more likely to have developed ovaries.

*Table S11*: The mass (in μg) of each hydrocarbon detected on the cuticles of 10 queens and 20 workers of *Bombus impatiens*.
